# The impact of multiple stenosis and aneurysms on arterial diseases: A cardiovascular study

**DOI:** 10.1016/j.heliyon.2024.e26889

**Published:** 2024-03-02

**Authors:** Mohammed Nasir Uddin, K.E. Hoque, M.M. Billah

**Affiliations:** a Department of Information and Communication Technology (ICT), Bangladesh University of Professionals (BUP), Dhaka-1216, Bangladesh; b Department of Arts and Sciences, Faculty of Engineering, Ahsanullah University of Science and Technology, Dhaka-1208, Bangladesh

**Keywords:** Stenosis artery, Aneurysmatic artery, Non-Newtonian, Finite element method, Blood flow

## Abstract

The comparative effect of serial stenosis and aneurysms arteries on blood flow is examined to identify atherosclerotic diseases. The finite element approach has been used to solve the continuity, momentum, and Oldroyd-B partial differential equations to analyze the blood flow. Newtonian and non-Newtonian both cases are taken for the viscoelastic response of blood. In this study, the impact of multiple stenotic and aneurysmal arteries on blood flow have been studied to determine the severity of atherosclerosis diseases through the analysis of blood behavior. The novel aspect of the study is its assessment of the severity of atherosclerotic disorders for the occurrence of serial stenosis and aneurysm simultaneously in the blood vessel wall in each of the four cases. The maximum abnormal arterial blood flow effect is found for the presence of serial stenoses compared to aneurysms which refers to the severity of atherosclerosis. At the hub of stenosis, the blood velocity magnitude and wall shear stress (WSS) are higher, whereas the arterial wall normal gradient values are lower. For all cases, the contrary results are observed at the hub of the aneurysmal model. The blood flow has been affected significantly by the increases in Reynolds number for both models. The influence of stenotic and aneurysmal arteries on blood flow is graphically illustrated in terms of the velocity profile, pressure distribution, and WSS. Medical experts may use this study's findings to assess the severity of cardiovascular diseases.

## 1 Introduction

Cardiovascular disease is one of the leading causes of mortality and morbidity in modern society. Stenosis or stricture is an irregular narrowing in a blood vessel [1]. It occurs due to the contraction of smooth muscle or reduces the space of the lumen which leads to atherosclerosis [2]. Aneurysm is well-defined as an extension of a blood vessel that occurs to abnormal progress or damaged blood vessel wall [3]. The most common symptoms of stenosis are hypertension, cardiovascular and coronary artery disease in the world [4] and peripheral, cerebral, and aortic aneurysmal diseases are found in the human blood vessels. The leading causes of stenosis and aneurysm are still an important subject of research and almost 60% [5] and 50% [6] of patients die before reaching the hospital because of the existence of narrow and widen arteries. At present, the growth of stenosis and aneurysm in blood vessels create serious circulation disorders and originate various arterial diseases. So, this sector has been drawing the attention of researchers recently [7,8]. Nowadays medical researchers, numerical scientists, and bioengineers’ effort jointly to provide blood simulation of the human circular system in normal and abnormal arteries in various situations. In human blood circulation, blood rheology with its elements has been changed for hemodynamic features. It performs a vital role in the progress and development of arterial diseases [9]. The numerical techniques, modeling of blood flow, and imaging procedures in bio-medical research can be joined simultaneously to increase accuracy in patient specification. It can play a significant role in increasing the accuracy and decreasing the expenses of training and therapy forecasts in the medical sector through data assimilation (DA) techniques and the availability of medical tools. Chung et al. [10] used DA methods to show blood flow simulation.

In the case of stenosis, researchers studied Newtonian fluid computationally, lab-based, and theoretically [11,12]. They showed that blood exhibits non-Newtonian activities due to shear-thinning, viscoelasticity, and thixotropy. At present, a few researchers [[13], [14], [15]] have been working on non-Newtonian fluids like blood flow having different conditions of stenosis. It is observed that the viscoelastic and shear thinning features of blood flow in nonuniform arteries contribute an important contribution to the basic knowledge and medication of arterial problems. Marshall et al. [16] analyzed the oscillatory blood flow in the case of stenosed carotid branching for MRI-based CFD which shows that cerebral strokes and myocardial infarction may occur because of the existence of constriction in the arteries. A blood simulation has been studied by Anand and Rajagopal [3]. They analyzed the Oldroyd-B fluid for their developed model. It is found that a thermodynamic framework is fitted for explaining the blood viscoelasticity behavior having several configurations [17]. D'Elia et al. [18] described the shear thinning conditions of blood by data assimilation technique for Navier-Stokes equations to show the blood simulation numerically in hemodynamics. Prokop and Kozel [19] studied the generalized Newtonian and Oldroyd-B types of fluid including an extended domain numerically. Achab et al. [20] studied oscillatory blood flow to determine the behavior of blood flow and the effect of the wall shear stress (WSS) on flow patterns in physiological requirements. The unsteady two-dimensional blood flows through a diseased artery featuring irregular stenosis has been analyzed theoretically and numerically by Tripathi et al. [21].

Recognized data has been used to improve the simulations [22] of blood flow in a two-dimensional Cartesian coordinate system for stenosis models. Uddin and Alim [23] studied the blood flows analyzed in uniform and non-uniform narrow and hardened arteries with different blood flows. Febina et al. [24] studied thoracic aortic aneurysms for finding out WSS and presented that the instruments are extremely useful as a medication methodology for cardiovascular problems. Vundla and Reddy [25] analyzed the blood flow with viscoelastic behavior in the fistula artery computationally. An analysis has been performed for blood flow with mild stenosis through the curved arteries by Kafle et al. [26]. Single and multiple sequential stenosis cases have been studied by Hoque et al. [2,27]. The authors identified the severity of atherosclerotic in a coronary artery using a noninvasive technique. Zaman and Khan [28] invested in the effects of blood fluid and curvature on unsteady blood flow in w-shaped constrained arteries. A stenosed mathematical model has been developed for blood flowing having post-stenotic dilatation and a force field [29]. Deep learning models [30] have been used for forecasting blood flow behavior in a double-stenosed artery.

In an aneurysm artery, researchers [6,31,32] studied the aneurysm with different perspectives and still have a large opportunity to know the key factors of the development of rupture and aneurysm in this area. It is important to understand the blood flow behavior through the bulging artery to detect arterial disease [12,[33], [34], [35]]. Finol and Amon [36] discussed the patterns of blood flow and hemodynamic stresses for abdominal aortic aneurysms of various sizes. A blood flow simulation has been discussed by Bernsdorf and Wang [37] for cerebral aneurysms and has shown the specific effect of the wall shear stress in the case of Newtonian and non-Newtonian fluids. Mukhopadhyay and Layek [38] analyzed blood flow through an aneurysm artery and showed the mixed impact of hematocrit and aneurysm on blood flow traits. An artificial neural network [6] has been used to predict mortality at hospitals after a ruptured abdominal aortic aneurysm. A computational fluid dynamics model [24] has been used for thoracic aortic aneurysms to assess the effect of wall shear stress on blood flow. They found the deteriorating WSS is an indicator in case of an aneurysm or rupture. A Fluid-Structure Interaction method [39] has been used to find out the effect of hematocrit behaviors on blood flow. They claimed that the dynamic behavior in a AAA and the variation of blood viscosity are related to hematocrit value. A study has been done on the role of the inflammatory response on the abdominal aortic aneurysm and vascular endothelial cells [40]. An analysis has been done with modeling and blood flow simulation for modified aneurysm models [41] to achieve the advanced forecast of hemodynamics.

Uddin et al. [42] examined the permeable aneurysmal effect on blood flow in human bodies. They found a remarkable change in the existence of permeability in aneurysmal arteries in the case of Newtonian and non-Newtonian fluids. A computational study has been performed to know the effect of blood flow characteristics due to the presence of rupture in the cerebral aneurysm by Shen et al. [43]. They observed that the incoming bloodstream with inclination is significantly effective in the aneurysmal area. Shen et al. [44] studied the blood flow simulation for the rupture of an aneurysm in the center cerebral artery.

With the above motivation, a mathematical analysis is done for cardiovascular study through the multiple stenosis and aneurysm arteries. Thus, an effort is made in the present theoretical study to determine the significant features of the blood flow through rigid multiple stenoses and aneurysmatic vessel. The generalized cross model is used to characterize the non-Newtonian blood flow behavior. In this study, the impact of multiple stenoses and aneurysmal arteries on blood flow characteristics have been investigated during artery narrowing and widening, including the WSS, the Reynolds number, and the Weissenberg numbers for Newtonian and Non-Newtonian flows. More focus is also paid to showing the major changes in cardiovascular diseases for the considered model. The impact of the multiple stenotic regions or aneurysmatic areas leads to identifying the severity of cardiovascular diseases. The impact of the wall shear stress on the blood flow at the stenotic region is a vital issue to identify atherosclerosis diseases which is another unique finding of the study. The following notations are used in mathematical structures. The dimensionless numbers Weissenberg number and Reynolds number are denoted by *Wi*, Re respectively, σ denotes stress tensor and P is the pressure. The velocity components are U and V along with X and Y respectively.

## 2 Physical model

**Fig. 1:**
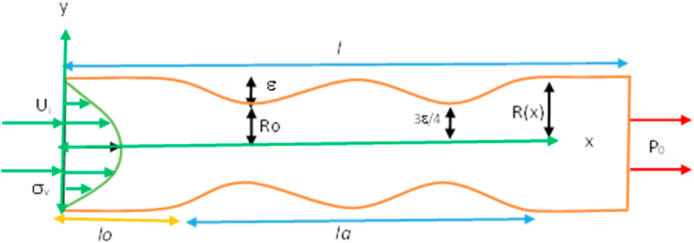
Computational domain with multiple stenoses.

**Fig. 2:**
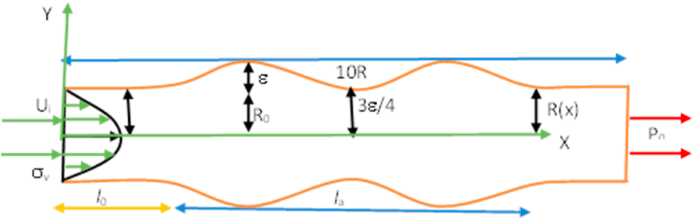
Geometry with multiple aneurysms arterial model.

**Fig. 3:**
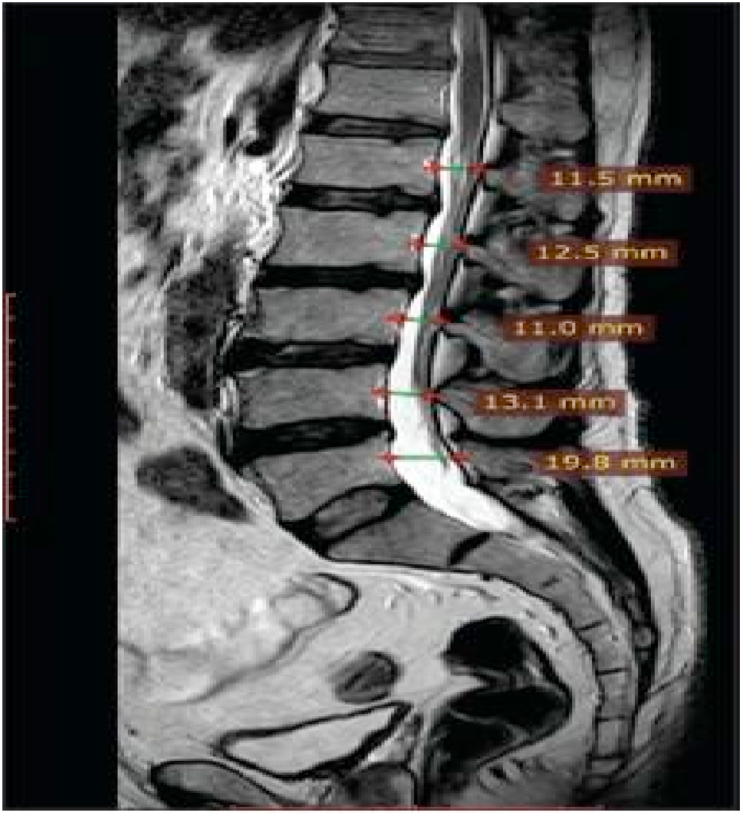
View of serial stenoses [45].

**Fig. 4:**
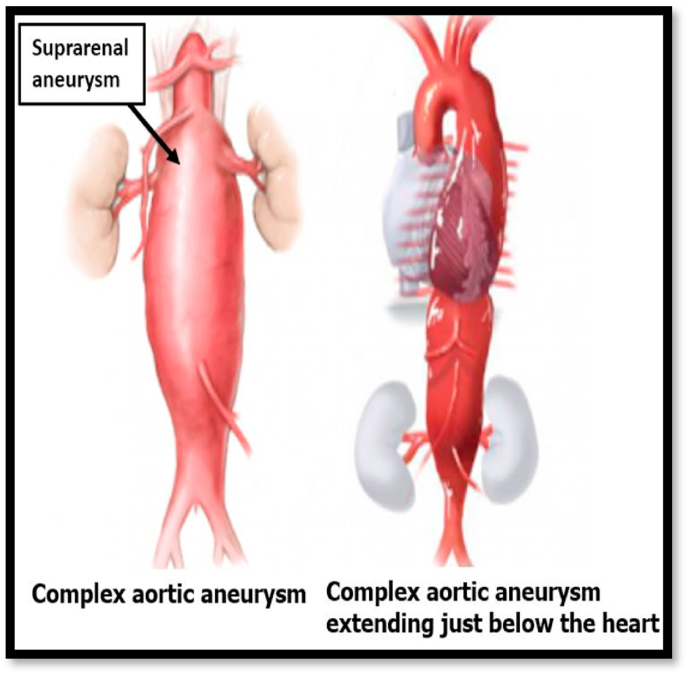
View of patient's multiple aneurysms [46].

In this study, the multiple stenosis and aneurysm artery models are analyzed using the Eulerian coordinate system. The computational domain of our target geometry is shown in Fig. 1, Fig. 2, however, Fig. 3, Fig. 4 are of key interest for considering it. It is supposed that the parabolic velocity profile and uniform pressure at the inlet and outlet respectively. The maximum blood velocity is found in the vessel's center, and the minimum is adjacent to the blood vessel wall. In this model, it is described by concentric layers of blood moving in parallel down the length of a blood vessel. In the case of laminar blood flow, the velocity distribution is parabolic at the inlet of the flow. On the other hand, uniform pressure is used at the outlet instead of an outflow condition for a better rate of convergence during iteration. All the blood vessel walls are considered adiabatic and inaccessible with no-slip conditions. To avoid the difficulty of the constitutive relations for both models, artery walls are assumed as inflexible.

In the stenosis model, Mathematical equations (1), (2) with a cosine shape [20] has been used. The stenotic artery's physical model and mathematical equation are as follows.

(1) $$y=\left\{ \begin{array}{c} 0.5D\left(1-\left(\varepsilon /D\right)\left(1+\cos\;\pi M\left(x\right)\right)\right);\;0\leq x\leq L_{s}\\ 1\;;\;\text{otherwise} \end{array}\right.$$

The development of the mathematical equation and geometrical model for the aneurysmal model is as below:

(2) $$h_{a}=\frac{R\left(x\right)}{R_{0}\left(x\right)}=\left\{ \begin{array}{c} 1+\frac{\varepsilon }{2R_{0}\;l_{a}^{4}}\left(11\left(x-l_{0}\right)l_{a}^{3}-47{\left(x-l_{0}\right)}^{2}l_{a}^{2}+72{\left(x-l_{0}\right)}^{3}l_{a}-36{\left(x-l_{0}\right)}^{4}\right);\;l_{0}\leq x\leq l_{0}+l_{a}\\ 1\;;\;\text{otherwise} \end{array}\right.$$

Where R(x) represents the radius of a normal section of the models, $R_{0}\left(x\right)$ denotes the radius of stenotic artery, *l*_*a*_ implies the length of stenotic or aneurysm area, and *l*_*0*_ is the location of stenotic or aneurysm, the highest aneurysm is ε. The positions of stenotic or aneurysmal are at $x=l_{0}+\frac{l_{a}}{6}\;\text{and}\;x=l_{0}+\frac{5l_{a}}{6}$ respectively. $\frac{3\varepsilon }{4}$ is the critical height at $x=l_{0}+\frac{l_{a}}{2}$, from the origin.

## 3 Mathematical analysis

The blood flow is assumed laminar and incompressible with blood behavior. The generalized models are taken to investigate the blood properties. The governing mathematical equations (3)–(5) [[47], [48], [49]] of steady-state blood flow are considered in the vector form as follows:

Continuity equation

(3) ∇.u=0

Momentum equation

(4) $$\rho \frac{\partial u}{\partial t}+\rho \left(u.\nabla \right)u=-\nabla p+\mu_{n}\Delta u+\nabla .\sigma +\rho \;f$$

Oldroyd-B equation

(5) $$\sigma +\lambda_{x}\left[\frac{\partial \sigma }{\partial t}+\left(u.\nabla \right)\sigma \right]=2\mu_{v}V\left(u\right)+\lambda_{x}[\sigma V^{/}-V^{/}\sigma -\sigma V-V\sigma ]$$

Here, velocity vector is ***u***, blood density, ρ = 1050 km/cm^3^*,* fluid stress tensor, σ *=*2 μ***V***, μ is the dynamic viscosity, λ_*x*_ is the relaxation and the strain rate tensor equation (6) [50].

(6) $$V=\frac{1}{2}\left(\nabla u+\nabla u^{T}\right)$$

The shear rate of blood flow is defined by $\dot{\gamma }=2\sqrt{V:V}=2\left|V\right|$ and it depends on viscosity function $\mu \left(\dot{\gamma }\right)$. The generalized Cross model equation (7) [[51], [52], [53]] is used for viscosity function as follows

(7) $$\mu \left(\dot{\gamma }\right)=\mu_{\infty }+\frac{\mu_{0}-\mu_{\infty }}{{\left(1+{\left(\lambda \dot{\gamma }\right)}^{b}\right)}^{a}}$$

Where, a, b, λ > 0.

The following dimensionless scales equation (8) [54] are considered to drive the non-dimensional equations for stenotic and aneurysmal models in the domain Ω:

(8) $$x*=x/L,t*=t\;U/L,U*=u/U,\sigma \;*=\sigma \;L/\;U\mu ,p*=\text{pL}\;/\mu U,f*=f\;L^{2}\;/\;\mu U,\nabla^{*}=\nabla L$$

Applying the above-mentioned non-dimensional variables into equations (3), (4), (5), we get the dimensionless governing mathematical equations:

Continuity equation

(9) ∇.U=0

Momentum equation

(10) Re[(U.∇)U]=−∇p+(1−)ΔU+∇.σ+f

Oldroyd-B equation:

(11) $$W_{i}\left[\left(U.\nabla \right)\sigma \right]+\sigma =2\mu_{v}\;V\left(U\right)+W_{i}\left[\left(\nabla U\right)\right]\sigma +\sigma \;{\left(\nabla U\right)}^{t}$$

Here, μ_v_ is viscoelasticity components, Newtonian viscosity components is μ_n_ and the relation, μ = μ_n_ + μ_v_ and $\frac{\lambda_{d}}{\lambda_{x}}=\frac{\mu_{n}}{\mu_{n}+\mu_{v}}$, where relaxation is λ_x_ and λ_d_ is retardation time. The fluid stress tensor, σ = σ_n_ + σ_v_, where σ_n_ = 2μ_n_**V** is Newtonian part and $\sigma_{v}+\lambda_{x}\frac{\partial \sigma_{v}}{\partial t}=2\mu_{v}V$ is viscoelastic part. The dimensionless numbers are Reynolds number (*Re*) and Weissenberg number (*Wi*).

It is essential to impose either Dirichlet velocity or Neumann surface force boundary restrictions [19] at inlet for velocity profile due to inadequate physiological data. The mathematical equations (12), (13) (more details [55]) for velocity and the corresponding components of fluid extra stresses are considered at inlet of the models

(12) $$U=1.5\;U_{i}\left(1‐y^{2}\right);\;0\leq y\leq 2,V=0,\sigma_{11}=2\mu_{v}Wi{\left(\frac{\partial U}{\partial y}\right)}^{2},{\;\sigma }_{12}=\mu_{v}\frac{\partial U}{\partial y},\sigma_{22}=0$$

At vessel walls, no slip conditions, **u** = 0 are applicable, and pressure remain constant at outlet. The extra stress equation is defined as follows for the vessel boundary with unit normal vector, **n**

(13) (σ.n).n=0

In this study, the non-dimensional partial differential equations (9), (10), (11) are considered for blood viscoelasticity (σ_v_) and shear-thinning (μ_n_) behavior. On the basis of μ_n_ and σ_v,_ the following cases are examined for various blood flow rates.

(i) Newtonian: μ_n_ = μ_∞_ = value of blood viscosity, σ_v_ = 0
(ii) Generalized Newtonian: μ_n_ = $\mu \left(\dot{\gamma }\right)$,σ_v_ = 0
(iii) Oldroyd-B: μ_n_ = μ_∞_ = value of blood viscosity, σ_v_
(iv) Generalized Oldroyd-B: μ_n_ = $\mu \left(\dot{\gamma }\right)$,σ_v_.

## 4 Numerical approach

### 4.1 Finite element analysis

Finite Element Method (FEM) [[56], [57], [58], [59]] is applied to solve the governing equations along with boundary conditions. The finite element equations have been developed using six-node triangular elements. These nodes correlated with velocities and stress tensors, but the pressure is associated with corner nodes. So, pressure is considered a lower-order polynomial function so that it satisfies the continuity equation. The velocity element, stress tensor and pressure distribution with linear interpolation [60] to their highest derivative orders in the governing equations (9)–(11) are classified as follows

(14) $$U\left(X,Y\right)=N_{\alpha }U_{\alpha },\sigma \left(X,Y\right)=N_{\alpha }\sigma_{\alpha },P\left(X,Y\right)=H_{\lambda }P_{\lambda }$$

Here, N_α_ is velocity interpolation functions, $\sigma_{\alpha }$ is stress interpolation function and the pressure interpolation function is H_λ_. equations (9)–(11) have transformed to the finite element equation using the weighted residuals technique then we get,

(15) $$\int_{A}N_{\alpha }\left(\nabla .U\right)\;dA=0$$

$$Re\int_{A}N_{\alpha }\left(U.\nabla )U\;\right)dA$$

(16) $$=-\int_{A}H_{\lambda }\nabla pdA+\int_{A}N_{\alpha }\left(\nabla .\sigma \right)\;dA+\left(1-\lambda \right)\int_{A}N_{\alpha }\Delta UdA+\int_{A}N_{\alpha }fdA$$

$$\int_{A}N_{\alpha }\left(U.\nabla \right)\;\sigma \;dA+\int_{A}N_{\alpha }\sigma dA$$

(17) $$=2\int_{A}\mu_{v}{\;N}_{\alpha }V\left(U\right)\;dA+W_{i}\int_{A}N_{\alpha }\left(\left(\nabla U\right)\sigma +\sigma^{t}\left(\nabla U\right)\;\right)dA$$

Where the element area is A. The boundary integral form has been produced after using Gauss's theorem for Equations (15)–(17) where the stress tensor and surface tractions are associated. So, we get the following equations (18), (19) [61],

$$Re\int_{A}N_{\alpha }\left(U.\nabla )U\;\right)dA+\int_{A}H_{\lambda }\nabla pdA-\int_{A}N_{\alpha }\left(\nabla .\sigma \right)dA-\left(1-\lambda \right)\int_{A}N_{\alpha }\Delta UdA-\int_{A}N_{\alpha }fdA$$

(18) $$=\int_{s_{0}}N_{\alpha }S_{x}ds_{0}$$

$$W_{i}\int_{A}N_{\alpha }\left(U.\nabla \right)\;\sigma \;dA+\int_{A}N_{\alpha }\sigma dA-2\int_{A}\mu_{v}{\;N}_{\alpha }V\left(U\right)\;dA-W_{i}\int_{A}N_{\alpha }\left(\left(\nabla U\right)\sigma +\sigma^{t}\left(\nabla U\right)\;\right)dA$$

(19) $$={\int_{A}N_{\alpha }\sigma }_{w}ds_{w}$$

Where the surface tractions (*S*_*x*_*, S*_*y*_) at outflow boundary *S*_*0*_ and the wall boundary *S*_*w*_ can be used to identifies the velocity and stress tensor components. The finite equation can be rewritten as follows after using equations (9), (10), (11),

(20) $$K_{\alpha \beta^{x}}u_{\beta }+K_{\alpha \beta^{y}}v_{\beta }=0$$

(21) $$Re\left(K_{\alpha \beta \gamma^{x}}u_{\beta }u_{\gamma }+K_{\alpha \beta \gamma^{y}}v_{\beta }u_{\gamma }\right)+M_{\alpha \mu^{x}}P\mu +K_{\alpha \beta^{x}}\sigma_{\beta }+K_{\alpha \beta^{y}}\sigma_{\beta }+\left(1-\lambda \right)\left(S_{\alpha \beta^{xx}}+S_{\alpha \beta^{yy}}\right)u_{\beta }-f_{x}K_{\alpha }=Q_{\alpha^{u}}$$

(22) $$W_{i}\left(K_{\alpha \beta \gamma^{x}}{\;u}_{\beta }\sigma_{\gamma }+K_{\alpha \beta \gamma^{y}}v_{\beta }\sigma_{\gamma }\right)+K_{\alpha \beta }\sigma_{\mu }-\mu_{v}\left(K_{\alpha \beta^{x}}u_{\alpha }+K_{\alpha \beta^{y}}u_{\beta }\right)\;-W_{i}\left(K_{\alpha \beta \gamma^{x}}u_{\beta }\sigma_{\gamma }+K_{\alpha \beta \gamma^{y}}u_{\beta }\sigma_{\gamma }\right)=Q_{\alpha^{T}}$$

Where,

$$K_{\alpha \beta^{x}}=\int_{A}N_{\alpha }N_{\beta ,x}dA,K_{\alpha \beta^{y}}=\int_{A}N_{\alpha }N_{\beta ,y}dA,K_{\alpha \beta \gamma^{x}}=\int_{A}N_{\alpha }N_{\beta }N_{\gamma ,x}dA\text{,}$$

$$K_{\alpha \beta \gamma^{y}}=\int_{A}N_{\alpha }N_{\beta }N_{\gamma ,y}dA,K_{\alpha \beta }=\int_{A}N_{\alpha }N_{\beta }dA,S_{\alpha \beta^{xx}}=\int_{A}N_{\alpha ,x}N_{\beta ,x}dA\text{,}$$

$$S_{\alpha \beta^{yy}}=\int_{A}N_{\alpha ,y}N_{\beta ,y}dA,M_{\alpha \mu^{x}}=\int_{A}H_{\alpha }H_{\mu ,x}dA,M_{\alpha \mu^{y}}=\int_{A}H_{\alpha }H_{\mu ,y}dA\text{,}$$

$$Q_{\alpha^{u}}=\int_{S_{0}}N_{\alpha }S_{x}dS_{0},Q_{\alpha^{T}}=\int_{S_{0}}N_{\alpha }\sigma_{w}dS_{w}$$

The details derivation of the element matrices is omitted for brevity and closed form solution is used for numerical analysis [[62], [63], [64]]. The set of non-linear algebraic equations (20)–(22) are solved by using reduced Newton's integration technique [65]. The PDE solver COMSOL Multiphysics [66] and MATLAB programing [67] are used to adapt the technique.

The following criteria of equation (23) [68] are used to ensure the convergence of the algorithm for all dependent variables in domain Ω

(23) $$\sum \left|{\Phi \;}_{ij}^{n}-{\Phi \;}_{ij}^{n-1}\right|\leq 10^{-5}$$

Where, Φ denotes a velocity, pressure and stress tensor variable *u, p*, σ respectively and *i, j* implies a nodal point of elements; and the number of iterations is *n* at the grid level.

### 4.2 Code validation

**Fig. 5(a):**
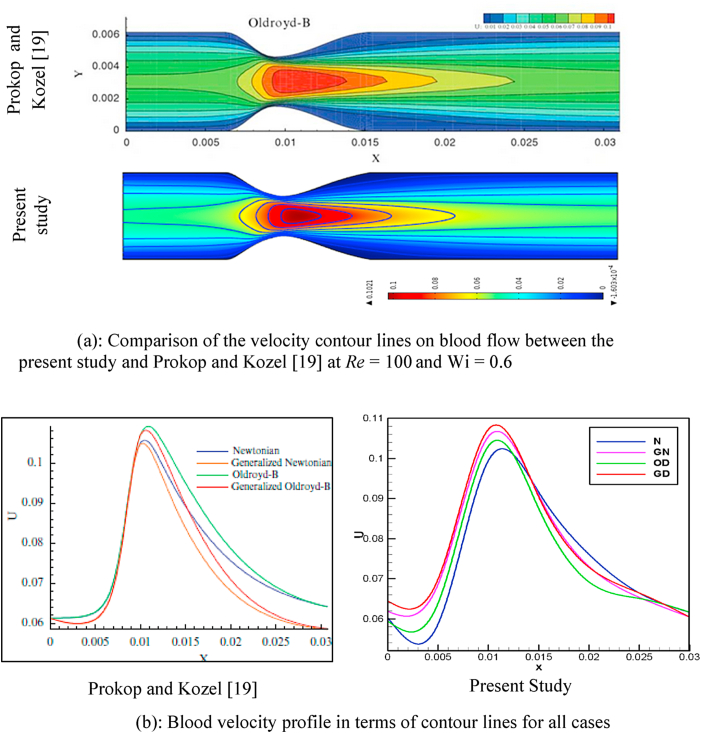
Comparison of the velocity contour lines on blood flow between the present study and Prokop and Kozel [19] at Re = 100 and *Wi* = 0.6 Fig. 5(b): Blood velocity profile in terms of contour lines for all cases.

The current numerical study has been validated with the published numerical work of Prokop and Kozel [19]. A single stenotic artery has been considered by Prokop and Kozel to study the numerical simulation of blood flow. Similar simulation parameters are used in the present study of the multiple stenoses and aneurysmatic arteries for the analysis of cardiovascular diseases. A validation test has been done to evaluate the accuracy of the numerical simulation and flow visualization for Newtonian (N), generalized Newtonian (GN), Oldroyd-B (OD), and generalized Oldroyd-B (GD) cases. It has computed blood velocity, pressure, and wall shear stress with dimensionless numbers *Wi* and Re for considered model. The blood flow simulation of the velocity field for Newtonian model has been examined and presented in Fig. 5(a). Additionally, Fig. 5(b) graphically displays the blood flow patterns for all four cases under consideration for code validation. At the stenotic Area of the artery, the maximum deviations are 4.99%, 8.57%, 6.13%, and 1.2% for Newtonian, Generalized Newtonian, Oldroyd-B, and Generalized Oldroyd-B models respectively. It is also found that the minimum deviations are 3.47%, 1.88%, 3.64%, and 1.08% for Newtonian, Generalized Newtonian, Oldroyd-B, and Generalized Oldroyd-B models respectively. The deviation is insignificant, and it may be a lack of smoothness in the geometry model. Fig. 5(a)-5(b) demonstrate excellent agreement with the previously published results of [19], proving the complete reliability of the present code and numerical approach.

## 5 Results discussion

In the work, the properties of blood thermal and tissue are used for the purpose of our simulation. We have estimated the velocity profile, pressure profile, and different Re numbers for the stenosis and aneurysmal models with various dimensionless numbers. The main goal of this work is to discuss the comparative impact of stenotic and aneurismatic models, WSS, and Reynolds numbers on the blood flow to identify the severity of atherosclerosis diseases for all cases. A comparative analysis has been done for both the cases of Newtonian and non-Newtonian through the stenotic and aneurysmal models. All cases are taken to describe the impact of viscoelastic and the shear-thinning behavior of blood. In the study, the following simulation parameters [69] are used: 0 ≤ W_i_ ≤ 1, 0 < *Re* ≤ 3000, μ_0_ = 0.16 [Pa.s], μ_n_ = 0.0036 [Pa.s], a = 1.23, b = 0.64, λ = 8.2 [s], ρ = 1050 [kg.m^−3^], T_b_ = 370 [c], C_b_ = 3770 [J/Kg.k]),

W_b_ = 0.5 [Kg/sec.m^3^], K = 0.5 [J/s.m.k], P_f_ = 400.

### 5.1 Effects of stenotic and aneurysmal artery on blood flow

**Fig. 6:**
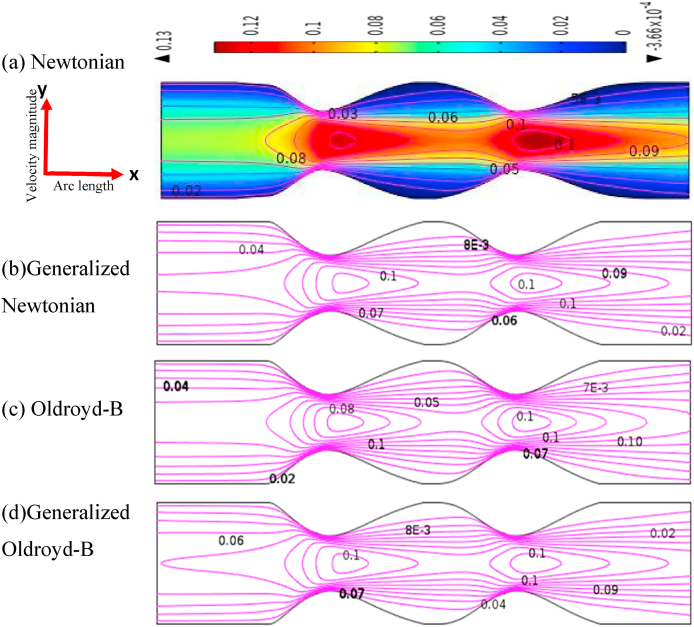
Blood velocity patterns of (a) Newtonian, (b) Non-Newtonian, (c) Oldroyd-B, and (d) Generalized Oldroyd-B for stenotic artery at R*e* = 1000 and *W*_*i*_ = 0.6.

**Fig. 7:**
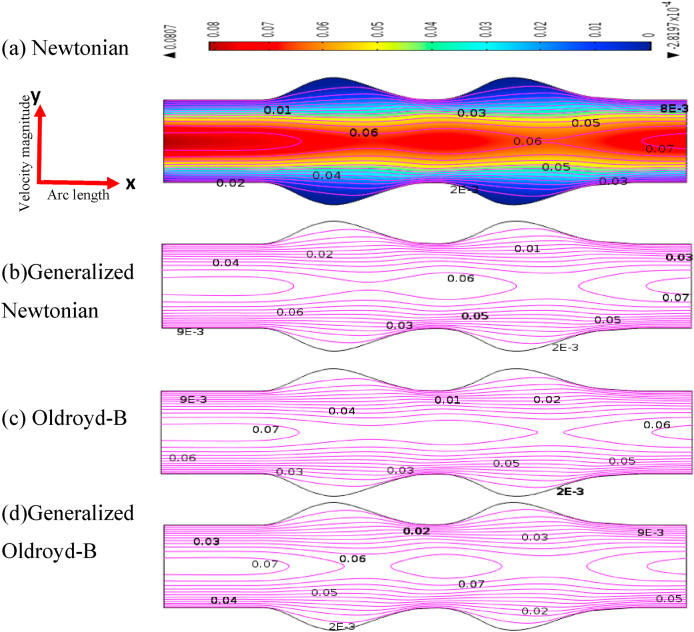
Blood velocity pattern (a) Newtonian, (b) non-Newtonian, (c) Oldroyd-B, and (d) Generalized Oldroyd-B for aneurysmatic artery at Re = 1000 and *W*_*i*_ = 0.6.

The blood flow simulation is shown in terms of velocity contour lines in Fig. 6, Fig. 7 of the stenosis and aneurysmal model for Newtonian and all non-Newtonian cases with uniform flow rates. In this study, we have discussed the effects of the stenotic model and aneurysmal model to find out the severity of atherosclerosis disease. It has given specific attention to identifying the model which affects atherosclerosis disease more. It is an important objective to identify the model which affects atherosclerosis disease more from this study.

Fig. 6, Fig. 7 show the velocity contour line comparisons of stenotic and aneurysmatic models for all four cases. It is interesting to note that there are permanent recirculation zones formed at the center of narrow regions for all cases. These recirculation zones are indicative of regions where the blood flow changes rapidly over a significant portion. Thus, the appearance of these recirculation regions is pathological significance since it increases the blood velocity which could be the cause of the constraint artery. The presence of recirculation zones in Fig. 6 leads to a higher velocity of blood flow which refers to the severity of atherosclerosis disease. This important factor of cardiovascular disease is found in the present study.

It is found that the blood flow has decreased more compared to the axis line at the adjacent artery wall immediately behind constrain areas in the stenotic model. As a result, reverse blood flows are observed in the area. For the generalized model and Oldroyd-B model, the recirculation is a little bit smaller due to the viscoelasticity and shear-thinning properties of blood. Atherosclerotic plaque produces disrupted blood flow in the artery which influences the overall circulation system of the human body.

On the other hand, in Fig. 7, an elliptical shape has been produced at the beginning of aneurysmal models and the blood flow has decreased rapidly at the swallow region for all cases. A little change is found in the blood velocity and the recirculation has originated for the generalized Oldroyd-B model only at the bulge area. The blood flow patterns have developed symmetrically with the vessel axis and the flow separation areas commenced from the beginning of the aneurysm. The lowest blood flow is seen adjacent to the aneurysmal wall which area indicates the weakness of the blood vessel wall. This vulnerable symptom of the vessel wall is one indicator of atherosclerosis disease, and it is one of the findings of this study.

**Fig. 8:**
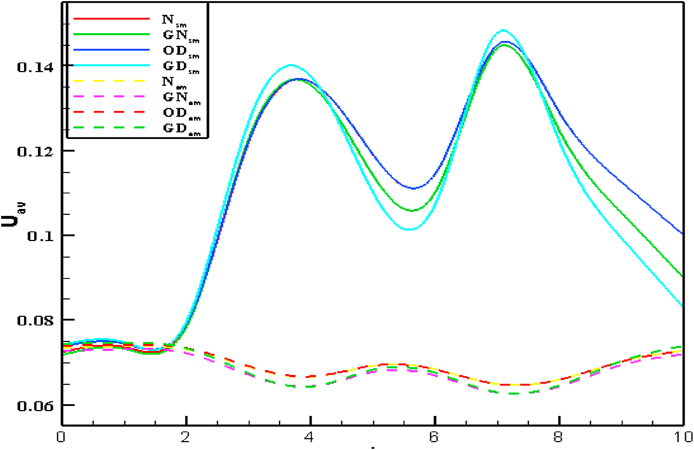
Blood velocity profile with stenotic and aneurysmal artery when Re = 1000 and *W*_*i*_ = 0.6.

The velocity profiles are shown graphically in Fig. 8 for all cases at *Wi* = 0.5 and Re = 1000 with the stenotic and aneurysmal model. The mathematical equation $\left|U\right|=\sqrt{u^{2}+v^{2}}$ is used to calculate velocity field. It is found that the velocity profiles are pronounced at and near the stenosis and similar results have been achieved by Achab's test [20]. In Fig. 6, the maximum velocity of almost 0.1478 is found for the generalized Oldroyd-B at the hub of the stenotic and the minimum blood velocity of 0.1074 (approximate) is between the two stenotic areas except for the inlet and outlet of the model. This result indicates the abnormality of blood flow due to blood clots or the development of stenosis in the blood vessel. More effects are observed for the generalized cases due to shear thinning or viscosity. The blood flow is highly affected by the generalized Oldroyd-B case for the stenosis model because of the presence of shear thinning and viscoelasticity characteristics. It is well known that blood is a non-Newtonian fluid. So, the generalized Oldroyd-B model provides more accurate results to analyze the blood flow compared to all others in the case of the stenosis model.

In the case of an aneurysm model, the velocity of blood flow is found nearly 0.069 for Newtonian and Oldroyd-B, 0.0685 for the generalized Oldroyd-B model and 0.068 for Oldroyd-B model in the middle of the two diastoles except for the entrance and exit which is shown in Fig. 8. The highest velocity has gained for the Newtonian case. The lowest blood velocity is approximately 0.0628 found at the 2nd diastole for generalized Newtonian and Oldroyd-B cases due to the use of the properties of shear-thinning or viscoelasticity. Therefore, the effect of the aneurysm model on blood flow is less compared to the stenosis model.

In the case of the stenosis model, the blood velocity has changed by 20.94%, 23.52%, 20.95%, and 27.33% for Newtonian, generalized Newtonian, Oldroyd-B, and generalized Oldroyd-B respectively whereas 3.19%, 8.32%, 7.19%, and 8.72% are found for aneurysm model. It conveys the message that blood pattern has affected more in a stenosis model compared to an aneurysmal model.

It is also observed that 23.52% and 27.33% have been found as the maximum changes at stenotic artery for generalized Newtonian and Oldroyd-B cases. But in the aneurysm model, only 8.32% and 8.72% velocity changes are found for the same cases. The highest deviation (27.33%) of blood velocity occurred for generalized Oldroyd-B cases at the throat of the stenotic artery due to the blood shear-thinning and viscoelasticity behavior. Consequently, the blood flow is more affected by the development of stenosis or plaque in the blood vessel which helps us to identify the severity of atherosclerosis diseases.

### 5.2 Effects of stenosis and aneurysmal artery on blood pressure distribution

**Fig. 9:**
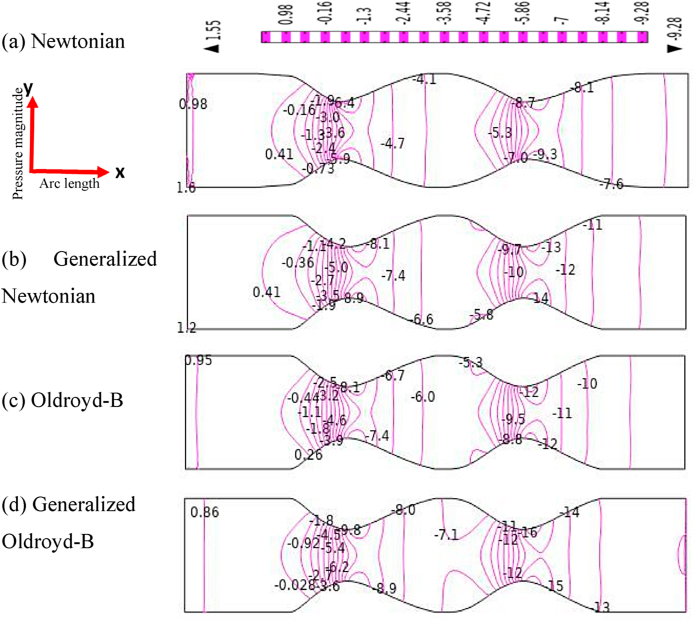
Blood pressure distribution (a) Newtonian, (b) non-Newtonian, (c) Oldroyd-B, and (d) Generalized Oldroyd-B for stenotic model at R*e* = 1000 and *Wi* = 0.6.

**Fig. 10:**
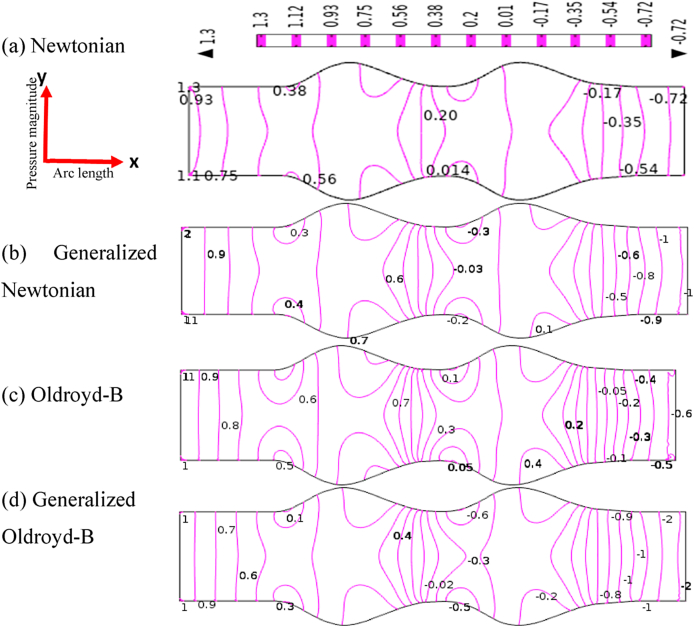
Blood pressure distribution (a) Newtonian, (b) Non-Newtonian, (c) Oldroyd-B, and (d) Generalized Oldroyd-B for aneurysmatic model R*e* = 1000 and *Wi* = 0.6.

The pressure contour lines of the blood flow simulation are exhibited in Fig. 9, Fig. 10 for both models. In this study, the models which are dominated to identify atherosclerosis diseases is one of the important findings. The blood pressure has developed axial profiles in front of the constraint area of the model for all cases. The pressure patterns are almost similar at the far of the stenotic area without little changes. The permanent parabolic pressure profiles have been created at the stenotic region due to a gain in low blood pressure for all cases. But just behind the stenosis area, the blood pressure patterns have originated some loops with the vessel walls. It is found that the pressure contour lines are quite steep at the nearest outlet of models and the pressure gradient is very intensive for the shear acting on the blood flow. In the presence of a stenotic artery in a blood vessel, the blood pressure has decreased rapidly, and the pressure has gained the highest value at the reattachment point which agrees with Muraki's test [70]. This finding may help to identify atherosclerosis disease in medical science. It is observed that the blood pressure patterns have changed more for the generalized cases and clear viscoelasticity and shear thinning effects are found for the generalized Oldroyd-B case. So, any stenotic area may bring important changes in blood pressure distribution in the human body which affect the whole circulation system.

In Fig. 10, the blood simulation is visible in terms of pressure contour lines for the aneurysmal model of all cases. The pressure patterns have produced some loops at the bulge region, and it has originated parabolic pressure profiles between the swell artery in the blood vessel because of the presence of narrowness of the artery model. The vertical contour lines are observed in front of the first aneurysm and at the end of the second aneurysm for all cases. The bottom-most blood pressure is noticed after the second bulge artery and the lowest blood pressure has been gained for Generalized Oldroyd-B. As blood flow passes the damaged or weaken blood vessels so blood particles may be clots or particles may die in bulge areas due to occluded regions of the blood vessels and loss of the natural distribution system of the human body. In this study, the changing signs of blood pressure profile are very significant outcomes to notify the diseases in the blood vessel artery.

**Fig. 11:**
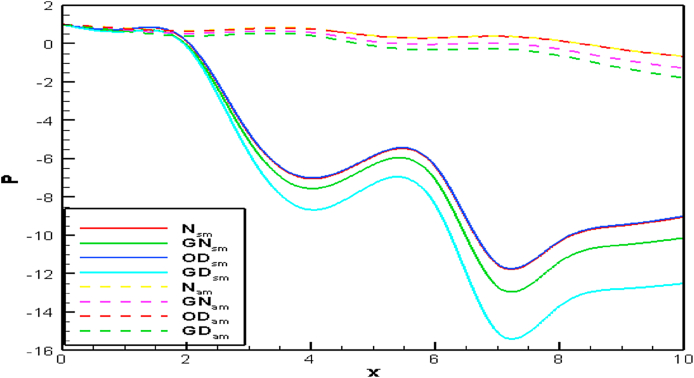
Compares of pressure profile for stenosis and aneurysm model along vessel axis when Re = 1000 and W_i_ = 0.6.

The blood pressure profiles are presented in Fig. 11 for both models graphically. In Fig. 11, the minimum and maximum values −14.25 and −5.11 are found respectively between two stenosis locations. On the contrary, the lowest and highest value −0.35 and 0.86 are observed in the confined area of diastole. These numerical values lead to blood pressure irregularities in the blood vessels of the human body for both stenotic and aneurysmatic artery conditions. The blood pressure profile has gone down more steeply than the aneurysm case and it gains the lowest value of −14.25 at the second stenotic for the generalized Oldroyd-B case but for the aneurysmal model, −0.35 is the lowest value for the same case at the second swell region. The pressure distribution changes between the stenotic are 50.97, 54.40, 50.97, and 51.16 percentages for Newtonian, generalized Newtonian, Oldroyd-B, and generalized Oldroyd-B respectively whereas 34.88, 84.50, 35.29, and 90 percentages are found for aneurysm model within two bulges area. Therefore, in the case stenotic model, blood pressure distribution has been affected by all cases by more than 50 percent, but the generalized cases have been influenced more by the aneurysmal model. Finally, it is found that the blood pressure distribution on blood flow has been disrupted for both models but high effects are visible for generalized cases.

### 5.3 Impacts of Reynolds Numbers on blood flow

**Fig. 12:**
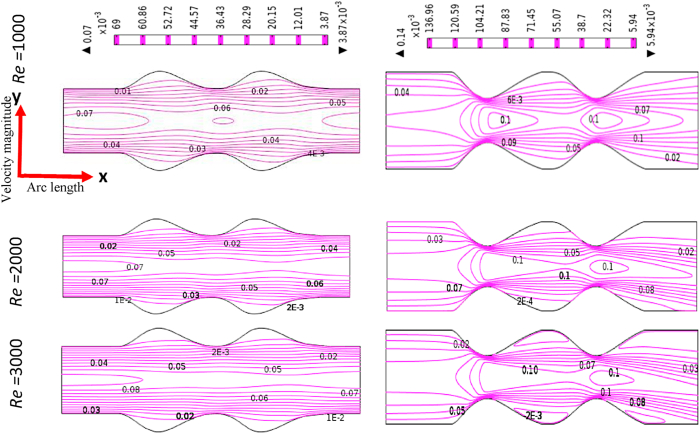
Effects of various Reynolds Numbers Re*=1000, 2000, and 3000* on Blood flow for both models at Newtonian.

**Fig. 13:**
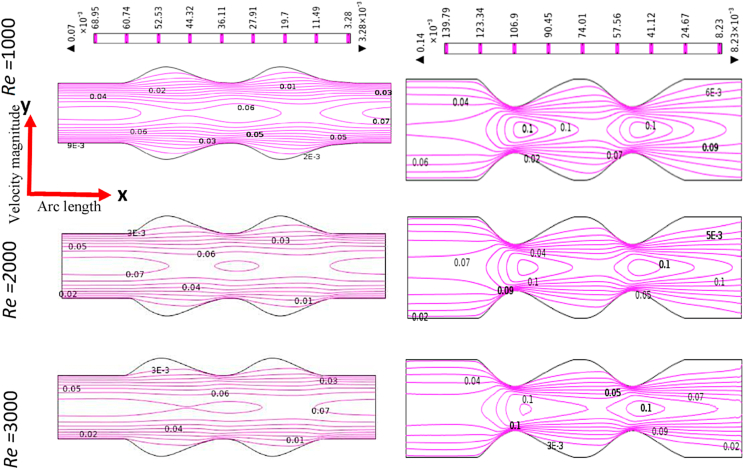
Effects of various Reynolds Numbers Re*=1000, 2000, and 3000* on Blood flow for both models at the generalized Newtonian case.

**Fig. 14:**
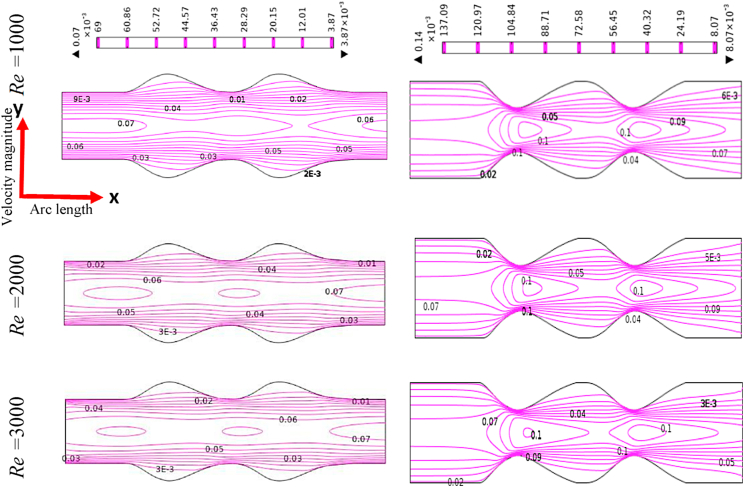
Effects of various Reynolds Numbers Re*=1000, 2000, and 3000* on Blood flow for both models at Oldroyd-B Model.

**Fig. 15:**
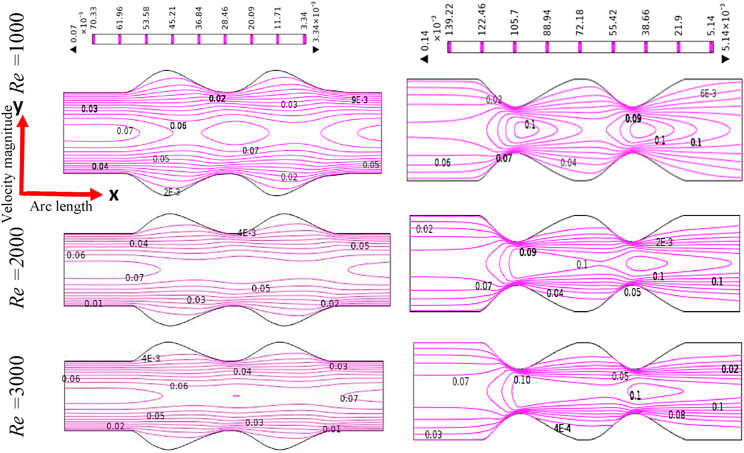
Effects of various Reynolds Numbers Re*=1000, 2000, and 3000* on Blood flow for both models at Generalized Oldroyd-B Model.

Fig. 12, Fig. 13, Fig. 14, Fig. 15 depict the blood simulation as contour lines with different Reynolds numbers (*Re*) are 1000, 2000, and 3000 for the stenosis and aneurysm models, respectively. In the stenotic and aneurysmatic models, it is found that the blood flow varies in all cases as Re increases. There is some permanent recirculation of blood flow at the center of stenosis and little changes are occurred with respect to Re but fully disappear at the 1st stenosis region for generalized Oldroyd-B case at Re = 3000. With the changes of Re the blood turns more chaotic and reverse blood flow is found after stenotic for Re = 2000 and 3000 which means the blood flow has turned into turbulent flow for the higher values of Re. On the contrary, the larger blood recirculation zones originated in the aneurysm model with the increases of Re for all four cases which signify that the blood flow is lower than the stenotic model for various Re. The symmetric blood flow patterns are observed at Re = 2000, but they are more impacted by the stenotic model. At Re = 3000, the blood flow behavior has altered slowly for the aneurysm model, but it has shifted rapidly for the stenosis model. In all circumstances, increased Re had a greater effect on blood flow in the stenotic model compared to the aneurysm model.

**Fig. 16(a):**
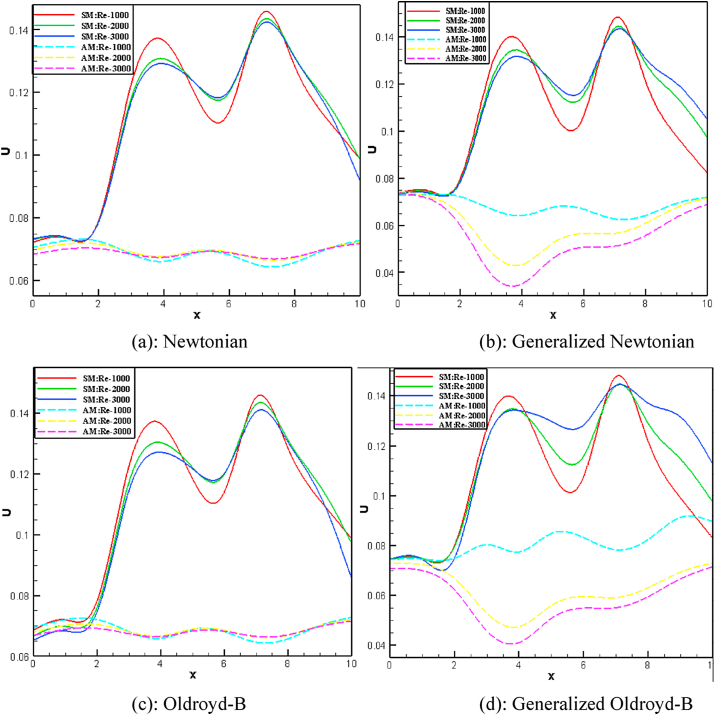
Newtonian Fig. 16(b): Generalized Newtonian Fig. 16(c): Oldroyd-B Fig. 16(d): Generalized Oldroyd-B Fig. 16: Velocity profile with the various Re on Blood flow for stenosis model (SM) and Aneurysmal model (AM) at W_i_ = 0.6 for all four cases.

The average blood velocity at the stenotic and aneurysm artery model is shown in Fig. 16 (a)–(d) graphically with various Re. The mean velocity has varied between two stenotic or aneurysm regions with the changes of Re. The maximum velocities of 0.152 and 0.072 are found for the stenotic and aneurysm models respectively. So, almost 52.63% higher velocity is gained at the stenotic model. The lowest velocities 0.133 and 0.056 are observed for the stenotic and aneurysm models respectively and 57.89% of blood velocity has deviated. It is clearly indicated that the development of stenosis in the artery is more harmful compared to the aneurysm artery. The blood velocity lines have crossed each other in the middle of two stenoses or aneurysms which leads to the turbulence of blood flow with the increases of Re.

### 5.4 Effects of Reynolds Numbers on blood pressure distribution

**Fig. 17:**
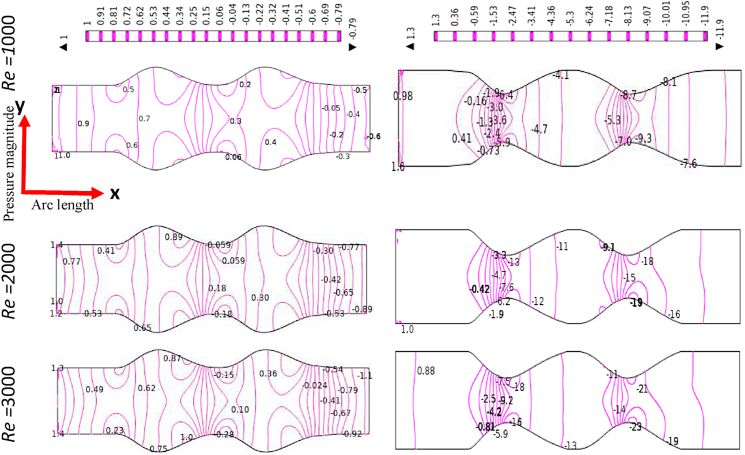
Pressure distribution of Blood flow with various Reynolds Numbers Re*=1000, 2000, and 3000* for Newtonian case.

**Fig. 18:**
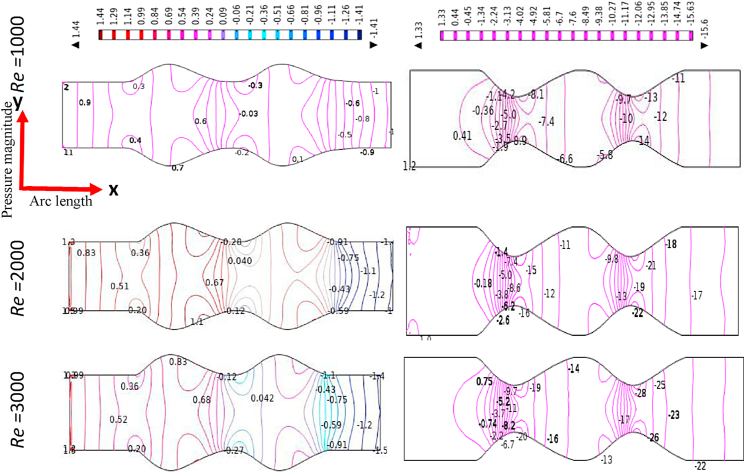
Pressure distribution of Blood flow with various Reynolds Numbers Re*=1000, 2000, and 3000* for Generalized Newtonian case.

**Fig. 19:**
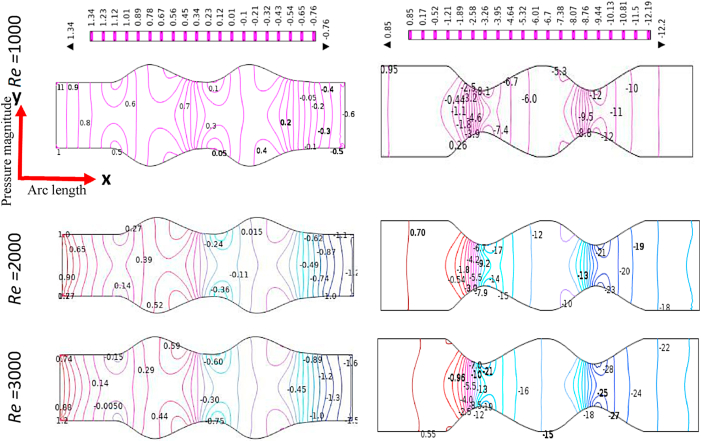
Pressure distribution of Blood flow with various Reynolds Numbers Re*=1000, 2000, and 3000* for Oldroyd-B case.

**Fig. 20:**
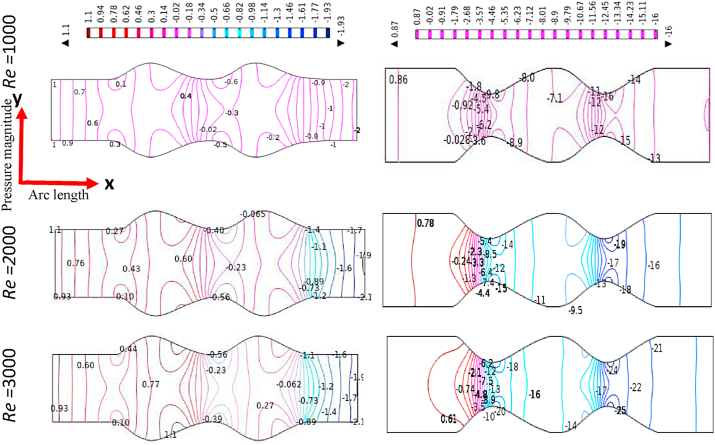
Pressure distribution of Blood flow with various Reynolds Numbers Re*=1000, 2000, and 3000* for Generalized Oldroyd-B Model.

The effect of Reynolds Numbers (*Re*) on blood pressure distribution is presented in Fig. 17, Fig. 18, Fig. 19, Fig. 20 for the Newtonian, Generalized Newtonian, Oldroyd-B, and Generalized Oldroyd-B. The pressure contour lines have been affected strongly by the increases of Re for both models at the stenotic and aneurysm region. The blood pressure profiles have decreased with the increase of Re and it is theoretically true for both models. The pressure decreasing at the stenotic region with the increasing of Re and [71] have found the same result. At the adjacent of inlet and outlet, the pressure profiles are alike in the four cases which have gained minimum values at the separation point, but intensive pressure gradients are found at the constraint. The parabolic pressure profile has developed in front of stenosis due to the heavy fluid shear acting at the area. The reattachment points just after constrain area to the vessel wall leads to higher pressure values for the stenosis artery model. For the aneurysmal model, the pressure contour lines are almost parallel to each other at the beginning and end of the model. At the first and 2nd aneurysms, the reattachment points have originated, and the separation points have been produced in the middle of the aneurysmal region. It is noticed that the lower values and higher values are found at the separation and reattachment points respectively. At Re = 1, the changes in pressure distribution are very light compared to higher Re = 2000 and 3000 at the bulge region. The pressure contour lines have fluctuated more at the swelled area of the model at Re = 3000 which leads to turbulent flow created in this area. The shear-thinning effect is visible in the generalized Oldroyd-B model for the higher values of Re.

**Fig. 21(a):**
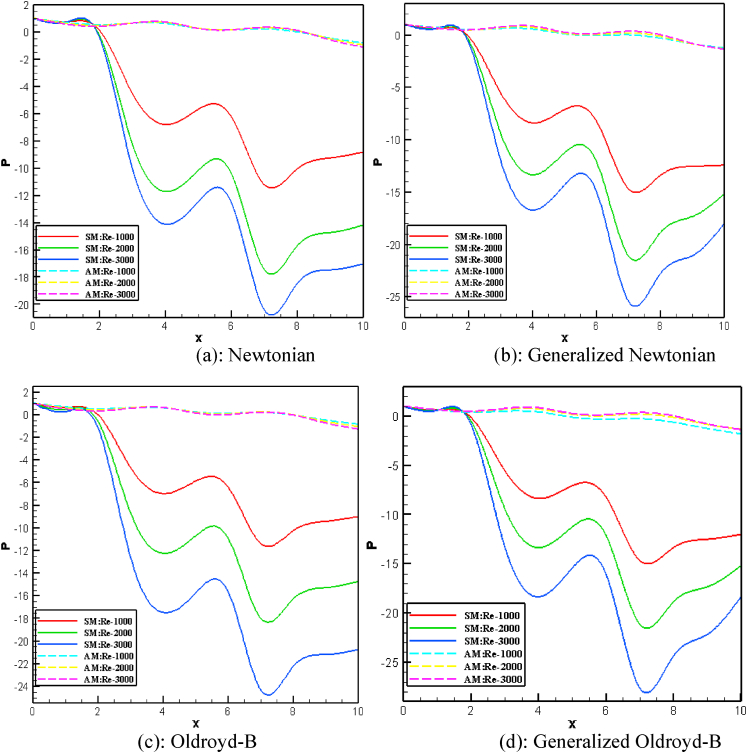
Newtonian Fig. 21(b): Generalized Newtonian Fig. 21(c): Oldroyd-B Fig. 21(d): Generalized Oldroyd-B Fig. 21: Pressure profile with the various Re on Blood flow for stenosis model (SM) and Aneurysmal model (AM) at W_i_ = 0.6 for all four cases.

Fig. 21(a) through 21(d) depict the pressure profiles for varied Re at *Wi* = 0.05. With the increase of *Re*, the blood pressure distribution has decreased in all cases for both models. The stenotic artery model has a greater impact on blood pressure distribution than the aneurysmal artery model.

The lowest blood pressure is −24.32 and 0.1696 for stenotic and aneurysm models respectively in the nearest area of plaque or bulge. The blood pressure has obtained the largest values 0.1451 and 0.7367 for stenotic and aneurysmal arteries respectively. So, the impact of the stenosis model on blood pressure is more severe compared to the aneurysm model. The blood pressure profiles have created turbulent flow in the stenotic area at a high Re number for generalized cases. In the aneurysm artery model, the blood pressure has changed mildly, and it is less harmful to atherosclerosis disease. On the other hand, a higher effect is found in blood vessels for developing plaque or hardening of the arteries.

### 5.5 The impact of wall shear stress on blood flow distribution

**Fig. 22:**
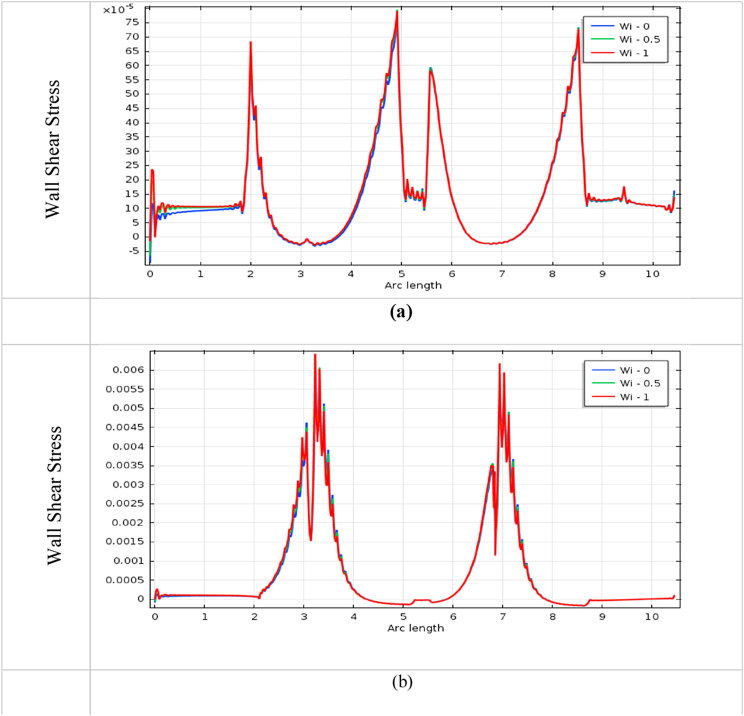
Impact of wall shear stress on blood flow for (a) aneurysmal and (b) stenotic artery at Newtonian case with various *Wi*.

**Fig. 23:**
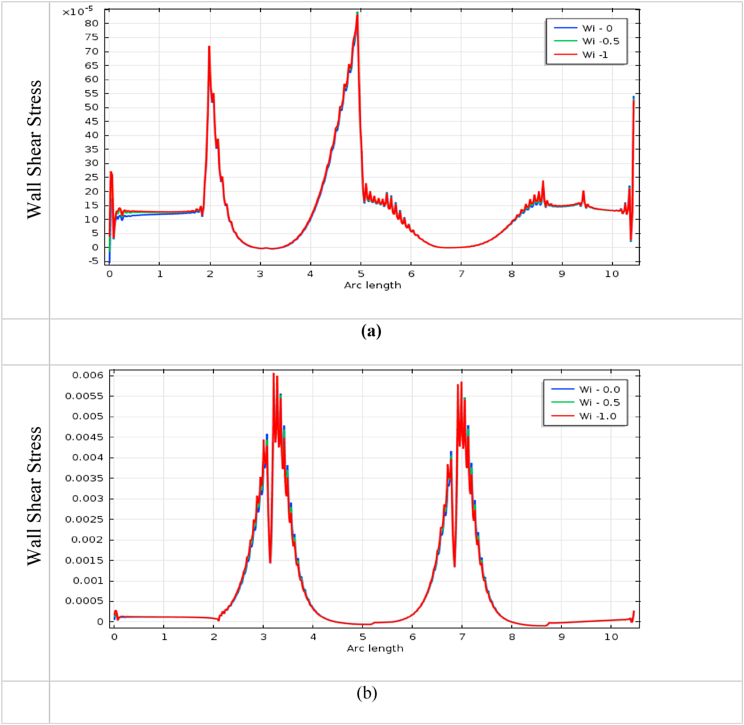
Impact of wall shear stress on blood flow for (a) aneurysmal and (b) stenotic artery at Generalized Newtonian case with various *W*_*i*_.

**Fig. 24:**
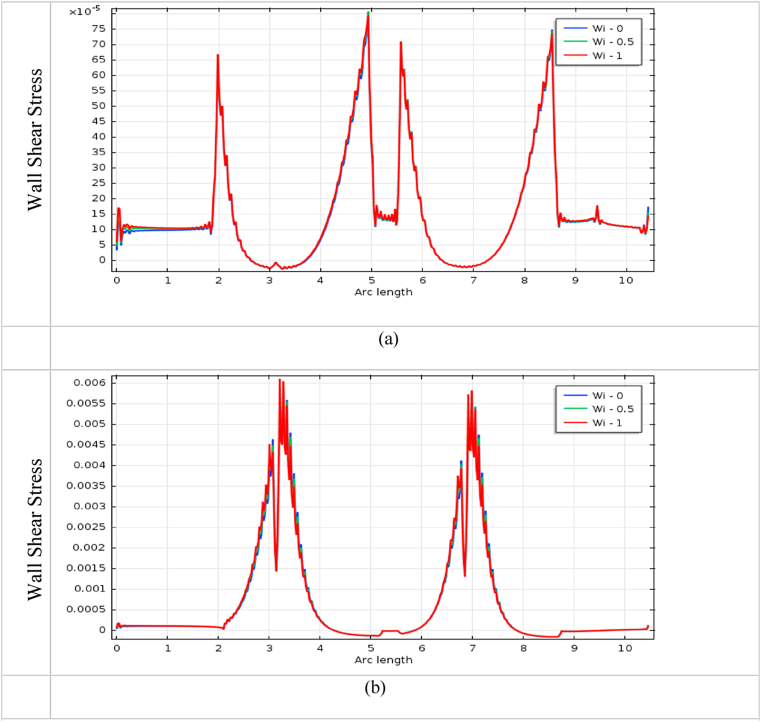
Impact of wall shear stress on blood flow for (a) aneurysmal and (b) stenotic artery at Oldroyd-B Model case with various *W*_*i*_.

**Fig. 25:**
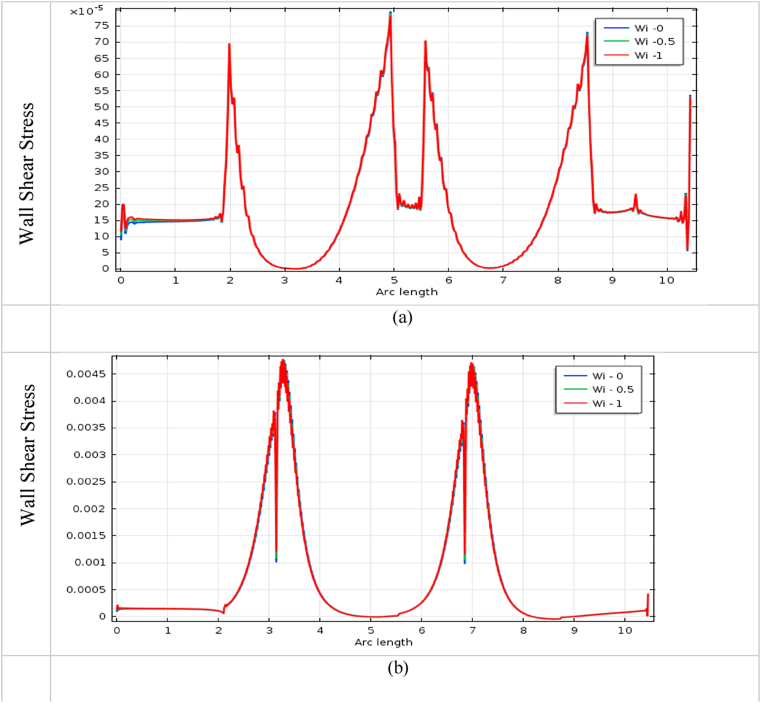
Impact of wall shear stress on blood flow for (a) aneurysmal and (b) stenotic artery at Generalized Oldroyd-B Model with various *W*_*i*_.

The impact of blood vessel WSS on blood flow is a vital indicator to finding the fatality of atherosclerosis disease in the arteries. It also plays an important role in understanding the commencement and development of cardiovascular diseases [72]. The blood flow has affected more at the throat of stenosis and aneurysm, and it is shown in Fig. 22, Fig. 23, Fig. 24, Fig. 25 for various *Wi* at the bottom wall with uniform blood flow rate. The wall surface forces have acted tangentially at the blood vessel arteries and worked against the blood fluid flow whose mathematical expression is WSS=(σ.n).t, Here n is the normal vector at the local wall and t is the corresponding unit tangential vector.

The measuring WSS provides information about the flow of the fluid near the blood vessel wall and it is an important topic of research in fluid dynamics. It is found in Fig. 22 (b), 23 (b), 24(b), and 25(b) that the effect of WSS is intense at the stenotic area and is mild at the diastole region for all cases. The WSS of the generalized Newtonian and Oldroyd-B model is slightly lower than the other models. The highest WSS effects are found in the stenotic artery model in the case of Newtonian. The minimum impacts of WSS are observed in the middle of two stenoses. The effect of wall shear stress on the stenotic artery is almost the same as Singh's result [73]. In Fig. 22 (a), 23 (a), 24(a), and 25(a), the lowest value is observed at the swell zone, and the greatest value is obtained at the center of the aneurysm of the artery. The value of WSS is the vice visa of the model of stenosis and aneurysmal model which means the model has worked properly. With the increases in *Wi*, the WSS has increased for both models. At the aneurysm model, the WSS speeds up sharply at the commencement of dilation and goes down at vasodilation and it gives negative values in this area for all cases. These negative values imply the presence of reversal blood flow in the aneurysm positions. For the stenosis model, WSS has increased dramatically at the hub of the constriction region and decreased at the non-stenosis area for all cases. The increased values of WSS are indicators of the presence of narrowing of the blood vessel artery and decreased values are inferred to be vasodilation area of the blood vessel.

## 6 Conclusions

The impact of multiple stenoses and aneurysms on arterial blood flow has been studied along with Newtonian and non-Newtonian cases. The Galerkin finite element method is used to simulate the problem. It is a significant outcome of this study that the non-Newtonian cases provide precise results compared to the Newtonian case to study blood flow behavior for stenosis and aneurysm models. Blood simulation and numerical analysis give a useful non-invasive technique to get real data on human blood flow in the blood vessel and proper determination of arterial diseases. The major findings of this study are as follows.

• The effect of serial aneurysms on blood flow is less harmful compared to multiple stenoses regions for all cases. Therefore, stenosis development is more detrimental to the human body, and it has led to noticeable alterations in conditions for normal arterial blood flow.
• The impact of serial stenoses on blood flow is the main root of the onset of arterial diseases.
• The impact of multiple aneurysms on blood vessels is another finding to determine atherosclerosis diseases.
• A stenotic model has created more impact on cardiovascular flow compared to an aneurysm model that helps to understand the severity of atherosclerosis diseases.
• The impact of the WSS on the constriction region is more significant to determine fetal atherosclerosis diseases.
• The blood flow behavior has been affected more with the increase of Reynolds number at the center of stenotic compared to the aneurysm regions.
• The maximum velocity and minimum pressure value have been obtained for the stenosis model compared to the aneurysm model for our all cases.
• The maximum WSS is found at the stenosis model compared to the aneurysm model which is an indicator of atherosclerosis diseases.

In this study, the two-dimensional models with the adiabatic and no-slip boundary conditions are considered. In real case of blood vessels are three-dimensional elastic vessels and it pumps blood around the body. To avoid the difficulty of the constitutive relations for both models, artery walls are assumed to be inflexible. We hope that we will overcome the limitations in our future works for applying the outcomes in a noninvasively way.

## Funding

None.

## Ethical approval

Not required.

## Data availability statement

The data that support the findings of this study will be available in **Kaggle** https://www.kaggle.com/datasets/drmohammednasiruddin/reynolds-numbers-dataset-for-analyzing-blood-flow/data from the date of publication, to allow for commercialisation of research findings.

## CRediT authorship contribution statement

**Mohammed Nasir Uddin:** Writing – review & editing, Software, Methodology, Investigation, Formal analysis, Data curation, Conceptualization. **K.E. Hoque:** Writing – original draft, Visualization, Software, Methodology, Formal analysis, Conceptualization. **M.M. Billah:** Writing – review & editing, Validation, Supervision, Methodology, Investigation, Formal analysis.

## Declaration of competing interest

The authors declare that they have no known competing financial interests or personal relationships that could have appeared to influence the work reported in this paper.

## Annex-1

### Transformation of momentum equation and Oldroyd-B equation

#### Conservation of momentum

The second principal physical law that fluid obeys is the conservation of momentum. This quantity is defined as the product of mass and velocity

$$\int_{v_{t}}\rho udx$$

Where ρ = ρ ***(x,t)*** and ***u = u(x,t).*** According to the second Newton's law, the rate of change of the momentum of the fluid contained in the volume ***v(t)*** is equal to all forces acting on this fluid. There are three types of forces acting on the fluid, body forces, surface forces and internal forces. The body force like gravity acts on a fluid particle and is proportional to its mass. It can be expressed as $\int_{v_{t}}\rho gdx$ where **g = g(x,t)** denotes the density of gravity force. The surface forces are the one acting on the boundaries *∂V(t)* of the fluid volume ***V(t)***, and is usually described by the stress tensor σ. The stress consists of pressure and friction forces, which are coming from the interactions between fluid layers sliding one relative to the other. Taking all together, conservation of momentum reads as

(24) $$\frac{d}{dt}\int_{v_{t}}\rho u=\int_{v_{t}}\rho f+\int_{\partial v_{t}}\sigma .n$$

Let J_t_ denote the Jacobian in the Eulerian frame. Then

(25) $$\frac{\partial }{\partial t}J_{t}\left(x\right)=\frac{\partial }{\partial t}J_{t}\left(t,x\right)=J_{t}\left(x)\nabla .u\left(t,x\right)=J\left(t,x\right)\nabla .u(t,L_{t}\left(e\right)\right)$$

Suppose *V*_*0*_*⊂ Ω*_*0*_ and *V*_*t*_*⊂Ω*_*t*_ be its image under the mapping *L*_*t*_. let us consider the function $f:I\times \Omega_{t}\to R$ be continuously differentiable with respect to both variables. Then,

(26) $$\frac{d}{dt}\int_{v_{t}}f=\int_{v_{t}}\left[\frac{Df}{Dt}+f\nabla .\;u\right]=\int_{v_{t}}\left[\frac{\partial f}{\partial t}+\nabla .\left(f\;u\right)\right]$$

The proof of this theorem can be found in Chorin and Marsden (2000).

Applying equations (1.1) and (1).2 then the transform of the above equation is

$$\frac{d}{dt}\int_{v_{t}}\rho u=\int_{v_{t}}\left[\frac{D}{Dt}\left(\rho u\right)+\rho u\nabla .u\right]=\int_{v_{t}}\rho \frac{D}{Dt}\left(u\right)$$

So the relation (1) can be written as

(27) $$\int_{v_{t}}\rho \frac{Du}{Dt}=\int_{v_{t}}\rho f+\int_{\partial v_{t}}\sigma .n$$

Applying the divergence theorem and assuming that *∇*.σ is integrable, the above relation (3) becomes

(28) $$\int_{v_{t}}\rho \frac{Du}{Dt}dx=\int_{v_{t}}\rho f+\int_{v_{t}}\nabla .\;\sigma$$

(29) $$\text{Which}\;\text{implies}\;\int_{v_{t}}\left(\rho \frac{Du}{Dt}-\nabla .\;\sigma -\rho f\right)=0$$

Since the volume *V*_*t*_ is arbitrary, with the hypothesis that the terms under the integrals are continuous in space, we derive the differential form of principle of linear momentum

(30) $$\rho \frac{Du}{Dt}-\nabla .\sigma =\rho fin\Omega_{t}$$

Writing the fluid acceleration $\frac{Du}{Dt}=\frac{\partial u}{\partial t}+\left(u.\nabla \right)u$, the relation (1.15–5) finally can be written as

(31) $$\rho \frac{\partial u}{\partial t}+\rho \left(u.\nabla \right)u-\nabla .\;\sigma =\rho \;f$$

**Oldroyd-B Equation:**

The Cauchy stress tensor is given by

$$\sigma =‐pI+\tau_{s}$$

In viscoelastic fluids, the stresses depend not only on the current motion of the fluid, but on the history of the motion. Where, extra-stress tensor, τ_s_ = σ_n_ + σ_v_

Here, $\sigma_{n}=2\mu \frac{\lambda_{d}}{\lambda_{x}}V\left(u\right),\text{with}\;\mu_{n}=\mu \frac{\lambda_{d}}{\lambda_{x}}$ the coefficient of Newtonian viscosity

Therefore, the Cauchy stress tensor can be written as

(32) $$\sigma =-pI+\sigma_{n}+\sigma_{v}=-pI+2\mu \frac{\lambda_{d}}{\lambda_{x}}V\left(u\right)+\sigma_{v}$$

Oldroyd observed that the convected time derivative $\frac{D\pi }{Dt}=\frac{\partial \pi }{\partial t}+\left(u.\nabla \right)\pi$ of a tensor π is not the objective and the objective form of the time derivative of a tensor can be written as

(33) $$\frac{D_{a}\pi }{Dt}=\frac{d\pi }{dt}+\pi V^{∕}\left(u\right)+{\left(\pi V^{∕}\left(u\right)\right)}^{t}-a\left[\pi V\left(u\right)+{\left(\pi V\left(u\right)\right)}^{t}\right]\;\Rightarrow \frac{D_{a}\pi }{Dt}=\frac{\partial \pi }{\partial t}+\left(u\;.\nabla \right)\pi +\pi V^{/}\left(u\right)-V^{∕}\left(u\right)\pi -a\left[\pi V\left(u\right)+V\left(u\right)\pi \right]$$

Where *-1 ≤ a ≤1* is a parameter.

Oldroyd suggested a general form of constitutive Equation by Hron (1997) as

(34) $$\lambda_{x}\frac{D_{a}\tau_{s}}{Dt}+\tau_{s}+\gamma \left(\tau_{s},\nabla u\right)=2\mu \left[\lambda_{d}\frac{D_{a}V\left(u\right)}{Dt}+V\left(u\right)\right],0\leq \lambda_{d}\leq \lambda_{x}$$

Consider *γ (τ*_*s*_*.∇****u****) = 0, λ*_*x*_*> (λ*_*d*_*> 0* and a = 1 for Oldroyd-B fluid then the general form of the constitutive equation can be express as

$$\lambda_{x}\frac{D_{a}\tau_{s}}{Dt}+\tau_{s}=2\mu \left[\lambda_{d}\frac{D_{a}V\left(u\right)}{Dt}+V\left(u\right)\right]\Rightarrow \lambda_{x}\frac{D_{a}\left(\sigma_{v}+\sigma_{n}\right)}{Dt}+\sigma_{v}+\sigma_{n}=2\mu \left[\lambda_{d}\frac{D_{a}V\left(u\right)}{Dt}+V\left(u\right)\right]\;\Rightarrow \lambda_{x}\frac{D_{a}\sigma_{v}}{Dt}+\lambda_{x}2\mu \frac{\lambda_{d}}{\lambda_{x}}\frac{D_{a}V\left(u\right)}{Dt}+\sigma_{v}+2\mu \frac{\lambda_{d}}{\lambda_{x}}V\left(u\right)=2\mu \left[\lambda_{d}\frac{D_{a}V\left(u\right)}{Dt}+V\left(u\right)\right]\;\Rightarrow \lambda_{x}\frac{D_{a}\sigma_{v}}{Dt}+\sigma_{v}=2\mu \left(1-\frac{\lambda_{d}}{\lambda_{x}}\right)V\left(u\right)\;\Rightarrow \lambda_{x}\frac{D_{a}\sigma_{v}}{Dt}+\sigma_{v}=2\left(\mu -\mu_{n}\right)V\left(u\right)\;\Rightarrow \lambda_{x}\frac{D_{a}\sigma_{v}}{Dt}+\sigma_{v}=2\mu_{v}V\left(u\right)$$

Where *μ*_*v*_*=μ- μ*_*n*_ is the coefficient of elastic viscosity and *μ=μ*_*v*_ *+ μ*_*n*_.

So, we have

(35) $$\lambda_{x}\frac{D_{a}\sigma_{v}}{Dt}+\sigma_{v}=2\mu_{v}V\left(u\right)$$

Finally, we can write by (9)

(36) $$\lambda_{x}\left[\frac{\partial \sigma_{v}}{\partial t}+\left(u\;.\nabla \right)\sigma_{v}\right]+\sigma_{v}=2\mu_{v}V\left(u\right)-\lambda_{x}\left[\sigma_{v}V^{/}\left(u\right)-V^{/}\left(u\right)\sigma_{v}-\sigma_{v}V\left(u\right)-V\left(u\right)\sigma_{v}\right]$$

Taking into account (8), the conservation law of momentum (7) can be written as follows

(37) $$\rho \frac{\partial u}{\partial t}+\rho \left(u.\nabla \right)u=\nabla .\sigma +\rho f=\nabla .\left[-pI+2\mu_{n}V\left(u\right)+\sigma_{v}\right]+\rho f=\nabla .\left(-pI\right)+\nabla .\left[2\mu_{n}[\nabla u+{\left(\nabla u\right)}^{t}\right]+\nabla .\;\sigma_{v}+\rho f=-p\nabla .I-\nabla p.I+\mu_{n}\nabla .\left(2V\left(u\right)\right)+\nabla .\sigma_{v}+\rho f=-\nabla p+2\mu_{n}\nabla .V\left(u\right)+\nabla .\sigma_{v}+\rho f$$

If *∇*.***u*** *= 0*, then we have *2∇.* ***V(u)****=Δ****u*.**

So, we can also write the conservation of momentum as

(38) $$\rho \frac{\partial u}{\partial t}+\rho \left(u.\nabla \right)u=-\nabla p+\mu_{n}\Delta u+\nabla .\sigma_{v}+\rho f$$

For simplicity, we write σ instead of σ_*v*_.

We have the system of non-linear equations formed by law of conservation of mass, the momentum equations and the Oldroyd-B constitutive equation as.

*∇*.***u****= 0*, in Ω

$$\rho \frac{\partial u}{\partial t}+\rho \left(u.\nabla \right)u=-\nabla p+\mu_{n}\Delta u+\nabla .\sigma_{v}+\rho f,in\Omega$$

$$\lambda_{x}\left[\frac{\partial \sigma }{\partial t}+\left(u\;.\nabla \right)\sigma \right]+\sigma =2\mu_{v}V\left(u\right)-\lambda_{x}\left[\sigma V^{/}\left(u\right)-V^{/}\left(u\right)\sigma -\sigma V\left(u\right)-V\left(u\right)\sigma \right],in\Omega$$

## References

[1] Krzeminski J.A., Baker L.M. (2006). Consumer health searcher. J. Consum. Health Internet.

[2] Hoque K.E., Ferdows M., Sawall S., Tzirtzilakis E.E., Xenos M.A. (2021). The impact of hemodynamic factors in a coronary main artery to detect the atherosclerotic severity: single and multiple sequential stenosis cases. Phys. Fluids.

[3] Anand M., R Rajagopal K. (2004). A shear-thinning viscoelastic fluid model for describing the flow of blood. Int. J. Cardiovasc. Med. Sci..

[4] Rajamannan N.M., Bonow R.O., Rahimtoola S.H. (2007). Calcific aortic stenosis: an update. Nat. Clin. Pract. Cardiovasc. Med..

[5] Taniguchi T. (2018). Sudden death in patients with severe aortic stenosis: observations from the CURRENT AS registry. J. Am. Heart Assoc..

[6] Wise E.S., Hocking K.M., Brophy C.M. (2015). Prediction of in-hospital mortality after ruptured abdominal aortic aneurysm repair using an artificial neural network. J. Vasc. Surg..

[7] Thurston G.B. (1973). Frequency and shear rate dependence of viscoelasticity of human blood. Biorheology.

[8] Fukushima T., Matsuzawa T., Homma T. (1989). Visualization and finite element analysis of pulsatile flow in models of the abdominal aortic aneurysm. Biorheology.

[9] Tu C., Deville M. (1996). Pulsatile flow of non-Newtonian fluids through arterial stenoses. J. Biomech..

[10] Chung C.E. (2010). Anthropogenic aerosol radiative forcing in Asia derived from regional models with atmospheric and aerosol data assimilation. Atmos. Chem. Phys..

[11] Nerem R.M. (1992). Vascular fluid mechanics, the arterial wall, and atherosclerosis. J. Biomech. Eng..

[12] Chakravarty S., Mandal P.K. (2000). Two-dimensional blood flow through tapered arteries under stenotic conditions. Int. J. Non Lin. Mech..

[13] Wang X., Li X. (2011). Computational simulation of aortic aneurysm using FSI method: influence of blood viscosity on aneurismal dynamic behaviors. Comput. Biol. Med..

[14] Hoque K.E., Ferdows M., Sawall S., Tzirtzilakis E.E., Xenos M.A. (2021). Hemodynamic characteristics expose the atherosclerotic severity in coronary main arteries: one-dimensional and three-dimensional approaches. Phys. Fluids.

[15] Tripathi J., Vasu B., Bég O.A., Gorla R.S.R., Kameswaran P.K. (2021). Computational simulation of rheological blood flow containing hybrid nanoparticles in an inclined catheterized artery with stenotic, aneurysmal and slip effects. Comput. Biol. Med..

[16] Marshall I., Zhao S., Papathanasopoulou P., Hoskins P., Xu X.Y. (2004). MRI and CFD studies of pulsatile flow in healthy and stenosed carotid bifurcation models. J. Biomech..

[17] Rajagopal K.R., Srinivasa A.R. (2011). A Gibbs-potential-based formulation for obtaining the response functions for a class of viscoelastic materials. Proc. R. Soc. A Math. Phys. Eng. Sci..

[18] D'Elia M., Perego M., Veneziani A. (2012). A variational data assimilation procedure for the incompressible Navier-Stokes equations in hemodynamics. J. Sci. Comput..

[19] Prokop V., Kozel K. (2013). Numerical simulation of generalized Newtonian and Oldroyd –B fluid. Numerical Mathematics and Advanced Applications.

[20] Achab L., Mahfoud M., Benhadid S. (2016). Numerical study of the non-Newtonian blood flow in a stenosed artery using two rheological models. Therm. Sci..

[21] Tripathi J., Vasu B., Bég O.A. (2021). Computational simulations of hybrid mediated nano- hemodynamics (Ag-Au/Blood) through an irregular symmetric stenosis. Comput. Biol. Med..

[22] Guerra T., Tiago J., Sequeira A. (2014). Optimal control in blood flow simulations. Int. J. Non Lin. Mech..

[23] Uddin M.N., Alim M.A. (2017). Numerical study of blood flow through symmetry and nonsymmetric stenosis artery under various flow rates. IOSR J. Dent. Med. Sci..

[24] Febina J., Sikkandar M.Y., Sudharsan N.M. (2018). Wall shear stress estimation of thoracic aortic aneurysm using computational fluid dynamics. Comput. Math. Methods Med..

[25] Vundla N., Reddy B.D. (2020).

[26] Kafle J., Gaire H.P., Pokhrel P.R., Kattel P. (2022). Analysis of blood flow through curved artery with mild stenosis. Math. Model. Comput..

[27] Zaman N., Ferdows M., Xenos M.A., Hoque K.E., Tzirtzilakis E.E. (2020). Effect of angle bifurcation and stenosis in coronary arteries: an idealized model study. BioMed Res. J..

[28] Zaman A., Khan A.A. (2021). Time dependent non-Newtonian nano-fluid (blood) flow in w-shape stenosed channel; with curvature effects. Math. Comput. Simul..

[29] Dhange M., Sankad G., Bhujakkanavar U. (2021). Blood flow with multiple stenoses in a force field. Math. Model. Eng. Probl..

[30] Jamil I.R., Humaira M. (2022). Modeling and predicting blood flow characteristics through double stenosed artery from computational fluid dynamics simulations using deep learning models. Int. J. Adv. Comput. Sci. Appl..

[31] Delucchi M., Spinner G.R., Scutari M., Bijlenga P., Morel S., Friedrich C.M., Furrer R., Hirsch S. (2022). Bayesian network analysis reveals the interplay of intracranial aneurysm rupture risk factors. Comput. Biol. Med..

[32] Jeong W., Rhee K. (2012). Hemodynamics of cerebral aneurysms: computational analyses of aneurysm progress and treatment. Comput. Math. Methods Med..

[33] Mistelbauer G. (2021). Semi-automatic vessel detection for challenging cases of peripheral arterial disease. Comput. Biol. Med..

[34] Güler İ., Übeyli E.D. (2005). Detection of ophthalmic arterial Doppler signals with Behcet disease using multilayer perceptron neural network. Comput. Biol. Med..

[35] Tu C., Deville M., Dheur L., Vanderschuren L. (1992). Finite element simulation of pulsatile flow through arterial stenosis. J. Biomech..

[36] Finol E.A., Amon C.H. (2001). Blood flow in abdominal aortic aneurysms: pulsatile flow hemodynamics. J. Biomech. Eng..

[37] Bernsdorf J., Wang D. (2009). Non-Newtonian blood flow simulation in cerebral aneurysms. Comput. Math. Appl..

[38] Mukhopadhyay S., Layek G.C. (2011). Analysis of blood flow through a modelled artery with an aneurysm. Appl. Math. Comput..

[39] Stergiou Y.G., Kanaris A.G., Mouza A.A., Paras S.V. (2019). Fluid-structure interaction in abdominal aortic aneurysms: effect of haematocrit. Fluids.

[40] Ramella M. (2019). Effect of cyclic stretch on vascular endothelial cells and abdominal aortic aneurysm (AAA): role in the inflammatory response. Int. J. Mol. Sci..

[41] Hong L.S., Hisham M.A., Adib M., Matalif M.U., Shafie Abdullah M., Mohd Taib N.H., Hassan R. (2020). Modeling and simulation of blood flow analysis on simplified aneurysm models. IOP Conf. Ser. Mater. Sci. Eng..

[42] Uddin M.N., Alim M.A., Karim M.M., Alam M.M. (2021). Effect of aneurysmatic artery on blood flow having permeability in human organ. J. Nav. Archit. Mar. Eng..

[43] Shen X.Y., Gerdroodbary M.B., Poozesh A., Musa Abazari A., Imani Sm S.M. (2021). Effects of blood flow characteristics on rupture of cerebral aneurysm: computational study. Int. J. Mod. Phys. C.

[44] Shen X.Y., Xu H.Q., Gerdroodbary M.B., Valiallah Mousavi S., Musa Abazari A., Imani S.M. (2022). Numerical simulation of blood flow effects on rupture of aneurysm in middle cerebral artery. Int. J. Mod. Phys. C.

[45] Kohli S., Kumar V., Narang S., Pawar I., Singhal A., Dalal V. (2014). Magnetic resonance imaging in the diagnosis of lumbar canal stenosis in Indian patients. J. Orthop. Allied Sci..

[46] Lobenwein D. (2022). Neuronal pre-and postconditioning via toll-like receptor 3 agonist or extracorporeal shock wave therapy as new treatment strategies for spinal cord ischemia: an in vitro study. J. Clin. Med..

[47] Warren R.B.B. (2006).

[48] Favero J.L., Secchi A.R., Cardozo N.S.M., Jasak H. (2010). Viscoelastic flow analysis using the software Open FOAM and differential constitutive equations. J. Nonnewton. Fluid Mech..

[49] Owens R.G., Phillips T.N. (2010).

[50] Batchlor G.K. (2000).

[51] Galidi A., Rannacher R., Turek S. (2008).

[52] Sequeira A., Janela J. (2007). A Portrait of State-Of-The-Art Research at the.

[53] Bodnár T., Sequeira A. (2008). Numerical simulation of the coagulation dynamics of blood. Comput. Math. Methods Med..

[54] Huba D. Joseph (2007).

[55] Bodnár T., Sequeira A., Prosi M. (2011). On the shear-thinning and viscoelastic effects of blood flow under various flow rates. Appl. Math. Comput..

[56] Connor J.J., Brebbia C.A. (1976).

[57] Uddin M.J., Rahman M.M. (2018). Finite element computational procedure for convective flow of nanofluids in an annulus. Therm. Sci. Eng. Prog..

[58] Sajjadinia S.S., Carpentieri B., Shriram D., Holzapfel G.A. (2022). Multi-fidelity surrogate modeling through hybrid machine learning for biomechanical and finite element analysis of soft tissues. Comput. Biol. Med..

[59] Hoque K.E., Ferdows M., Sawall S., Tzirtzilakis E.E. (2020). The effect of hemodynamic parameters in patient-based coronary artery models with serial stenoses: normal and hypertension cases. Comput. Methods Biomech. Biomed. Engin..

[60] Seshu P. (2012).

[61] Singiresu S. (2004).

[62] Saunders H. (1987). Finite element methods—an introduction. Finite Elem. Anal. Des..

[63] Yeasmin S., Islam Z., Azad A.K., Alam M.M., Rahman M.M., Karim M.F. (2022). Thermal performance of a hollow cylinder with low conductive materials in a lid-driven square cavity with partially cooled vertical wall. Therm. Sci. Eng. Prog..

[64] Uddin M.N., Uddin M.M., Alam M.M. (2022). Comparative mathematical study of blood flow through stenotic and aneurysmatic artery with the presence and absence of blood clots. Malaysian Journal of Mathematical Sciences.

[65] Zienkiewicz O.C., Taylor R.L., Too J.M. (1971). Reduced integration technique in general analysis of plates and shells. Int. J. Numer. Methods Eng..

[66] (2013). COMSOL Multiphysics.

[67] Attaway S. (2013).

[68] Querin Osvaldo M., Victoria Mariano, Alonso Cristina, Ansola Rubén, Martí Pascual (2017).

[69] Shih T.C., Yuan P., Lin W., Kou H.S. (2007). Analytical analysis of the Pennes bioheat transfer equation with sinusoidal heat flux condition on skin surface. Med. Eng. Phys..

[70] Muraki N. (1983). Ultrasonic studies of the abdominal aorta with special reference to hemodynamic considerations on thrombus formation in the abdominal aortic aneurysm. J. Japanese College Angiology.

[71] Donald F. (1973). Flow characteristics in models of arterial stenoses-I Steady flow. J. Biomech..

[72] Ferdows M., Hoque K.E., Bangalee M.Z.I., Xenos M.A. (2022). Wall shear stress indicators influence the regular hemodynamic conditions in coronary main arterial diseases: cardiovascular abnormalities. Comput. Methods Biomech. Biomed. Engin..

[73] Singha K., Shingh D.P. (2013). Effect of hematocrit on wall shear stress for blood flow through tapered artery. Appl. Bionics Biomech..

